# Divergence of Primary Cognate B- and T-Cell Proliferative Responses to Subcutaneous and Intravenous Immunization with Virus-Like Particles

**DOI:** 10.3390/v6083334

**Published:** 2014-08-22

**Authors:** Vladimir Temchura, Svetlana Kalinin, Ghulam Nabi, Bettina Tippler, Thomas Niezold, Klaus Überla

**Affiliations:** Department of Molecular and Medical Virology, Ruhr-University Bochum, Bochum, D-44780, Germany; E-Mails: svetlana.kalinin@ruhr-uni-bochum.de (S.K.); ghulam.nabi@ruhr-uni-bochum.de (G.N.); bettina.g.tippler@rub.de (B.T.); thomas.niezold@gmx.de (T.N.); klaus.ueberla@ruhr-uni-bochum.de (K.Ü.)

**Keywords:** lentiviral VLP, B-cell proliferation, T-cell proliferation

## Abstract

A major advantage of virus-like particle (VLP) vaccines against HIV is their structural identity to wild-type viruses, ensuring that antigen-specific B-cells encounter the envelope protein in its natural conformation. For the induction of affinity-matured antibodies, the B-cells must also obtain help from T-cells that are restricted by linear epitopes. Using B- and T-cell transgenic mouse models, we compared the efficacy of modified HIV-VLPs delivered by subcutaneous and intravenous immunization to stimulate primary B- and T-cell proliferative responses in different lymphoid organs. VLPs containing an influenza virus hemagglutinin epitope within the HIV-Gag protein induced comparable primary cognate T-cell proliferative responses in the draining lymph node and the spleen, irrespective of the delivery route. In contrast, after subcutaneous immunization with HIV-Gag VLPs containing hen egg lysozyme (HEL) on their surface, the proliferative response of transgenic HEL-specific B-cells was restricted to the draining lymph nodes, while intravenous VLP immunization primarily induced a B-cell proliferative response in the spleen. *In vitro* co-culture experiments further revealed that the presentation of VLP-associated surface antigens by dendritic cells to cognate B-cells is inefficient. This is consistent with a direct triggering of the B-cell proliferative response by the VLPs and suggests that HIV VLPs may indeed be suitable to directly promote the expansion of B-cells specific for conformational epitopes that are unique to functionally-active Env spikes on the virion. Further investigations are warranted to explore potential differences in the quality and protective potency of HIV-specific antibody responses induced by the two routes.

## 1. Introduction

High variability, the inaccessibility of functionally-important conserved epitopes and the conformational instability of the HIV Env protein constitute major hurdles for the development of Env-based vaccines inducing broadly-protective antibody responses (reviewed in [1]). In comparison to monomeric protein vaccines, virus-like particles (VLP) of HIV may have several advantageous properties. The particulate structure of the VLP and the repetitive nature of the Env protein on the surface of the VLP have been responsible for the more efficient induction of humoral and cellular immune responses. In addition, the Env protein can be incorporated into the VLP in a membrane-embedded form in its natural conformation. This conformation may facilitate the induction of antibodies against conformation-dependent epitopes that are unique to the trimeric Env spikes on the virion and may avoid distractive antibody responses to epitopes present only on the shed monomer. Mice vaccinated with HIV-VLPs raised antibodies that recognized a broader number of Env-specific peptides at higher titers than mice vaccinated with soluble envelope protein [2]. In our studies on SIV-VLPs, we also observed stronger antibody responses after immunization with VLPs containing membrane-bound gp130 of SIV in comparison to soluble gp130 [3]. Mice vaccinated with VLP were also reported to have antibodies that blocked viral infection of neutralization-resistant viruses in an *in vitro* neutralization assay [2]. Additionally, neutralizing responses against native HIV-Env trimers can be induced in different animal models after immunization with high doses of HIV-1 VLPs [4]. A prerequisite for the induction of such antibodies is that naive B-cells are indeed exposed to the Env spikes in their natural conformation in the B-cell areas of secondary lymphoid organs. Using fluorescently-labeled VLPs, Cubas and colleagues showed that after i.d. immunization, VLPs can enter lymph nodes in an intact form without disruption of their membranous envelope [5]. In the last decade, different mechanism for antigen entry into the secondary lymphoid organs were described (reviewed in [6,7,8]). VLPs may enter lymphoid follicles by diffusion via gaps in the floor of the subcapsular sinuses. They may also be actively transported into lymphoid organs by subcapsular sinus macrophages or migratory DCs (reviewed in [6,7,8]).

In addition to the antigen in its natural conformation, B-cells also require signals from T-helper cells for differentiation into memory B-cells and affinity maturation. The T-helper cells are primed by cognate interaction with activated DCs presenting antigen-derived peptides on MHC-II complexes and co-stimulatory molecules. This initial activation results in extensive proliferation and clonal expansion of antigen-specific CD4^+^ T-cells (reviewed in [9]). After differentiation into follicular T-helper cells, they can provide B-cell help and affinity maturation. We recently demonstrated that the T-helper cell function for the Env protein after immunization with HIV-VLPs is not restricted to Env-specific T-helper cells. Due to the particulate nature of HIV-VLPs, T-helper cells specific for the HIV GagPol protein were able to provide intrastructural help for Env-specific B-cells [10].

Thus, a vaccine aiming at the induction of a protective antibody response against HIV should trigger the activation and expansion of T-helper cells, requiring efficient uptake, processing and presentation of the antigens by DCs. At the same time, the vaccine needs to deliver the Env protein in its native conformation to the B-cell area of lymphoid organs. One of the earliest indicators of appropriate B- and T-cell stimulation detectable *in vivo* after vaccination is the proliferative response of antigen-specific B- and T-cells. To test whether VLPs can trigger both arms of the immune system, we employed very sensitive T-cell and B-cell receptor transgenic mouse models and compared the proliferative responses of cognate B- and T-cells in lymphatic tissues during the first week after subcutaneous and intravenous VLP immunization.

## 2. Materials and Methods

### 2.1. Mice

Mice were housed in singly-ventilated cages in the animal facility of the Faculty of Medicine, Ruhr University Bochum, Germany, in accordance with the national law and were handled according to instructions of the Federation of European Laboratory Animal Science Associations. Six- to eight-week-old female C57BL/6J (BL6) (Janvier, France), BALB/c (Charles River, Germany), mice with transgenic class II MHC-restricted T cell-receptor (TCR) specific for the hemagglutinin HA_110-120_ peptide (TCR-HA mice) (in-house breeding) and mice in which hen egg lysozyme (HEL)-specific B cells can switch to all Ig isotypes (SW-HEL mice) (in-house breeding) were used in this study. Approval for the animal experiments was obtained from the Landesamt für Natur, Umwelt und Verbraucherschutz Nordrhein-Westfalen.

### 2.2. Cell Lines, Plasmids, VLP Production and Characterization

HEK293T cells were cultured in Dulbecco’s modified Eagle Medium (DMEM) (Life Technologies, Carlsbad, CA, USA) with 10% fetal calf serum (FCS) (Life Technologies) and appropriate antibiotics. The plasmids, Hgpsyn (a codon-optimized HIV GagPol expression plasmid) [11], pConBgp140GCD (a codon-optimized HIV-Env clade B consensus sequence) [10] and pC-HEL-TM (encoding the membrane-anchored form of HEL) [12], have been described. The HIV-Gag expression plasmid was constructed from Hgpsyn by deleting the coding sequences for the amino acid sequences downstream of those encoding GNDPSSQ. Coding sequences for a T-helper cell epitope from influenza virus hemagglutinin, SFERFEIFPKE, flanked by alanines, were inserted between the codons for amino acid DTGHSSQ and VSQNYPI of Hgpsyn by overlap extension PCR. The resulting Hgpsyn-HA plasmid encoded the following amino acids between matrix and capsid: DTGHSSQASFERFEIFPKEAVSQNYPI. Pol sequences encoding amino acids from SLPGRW to ASRQDED were deleted from Hgpsyn-HA to generate Hgsyn-HA.

To produce VLPs, HEK293T cells were transiently co-transfected with pConBgp140GCD or pC-HEL-TM plasmid and one of the Gag or GagPol expression plasmids described above (Table 1) using polyethylenimine [10]. The medium was replaced 18 h after transfection with fresh AIM-V^®^ medium (Life Technologies), and cells were incubated for 48 hours. VLPs were concentrated by ultracentrifugation through a 20% sucrose cushion, as described [10]. The p24 content of the VLP preparations was determined with an anti-p24CA-ELISA, and western blot analyses for Gag were performed, as reported elsewhere [13]. The anti-HEL ELISA was described previously [12]. The endotoxin levels were measured by the QCL-1000^®^ Chromogenic LAL Endpoint Assay (Lonza, Walkersville, MD, USA).

### 2.3. Cell Isolation, Purification and CFSE Labeling

For *in vitro* experiments, CD11c^+^ DCs were enriched by positive selection with anti-CD11c magnetic beads (Miltenyi Biotec, Bergisch Gladbach, Germany) from a single-cell suspension of splenocytes from BL6 mice. Naive untouched B-cells were isolated from a single-cell suspension of splenocytes and lymph node (LN) cells from SW-HEL mice with the B-Cell Isolation Kit (Miltenyi Biotec). All isolations were done according to the manufacturer's instructions. The resulting cells were routinely >98% pure. For adoptive transfer, naive TCR-HA CD4^+^ T-cells and SW-HEL B-cells were isolated from a single-cell suspension of splenocytes and LN cells of the transgenic mouse strains. For *in vivo* and *in vitro* proliferation assays, cells were labeled with 5 µM Carboxyfluorescein succinimidyl ester (CFSE), as recommended by manufacturer (Vybrant CFDA Cell Tracer kit, Invitrogen, Carlsbad, CA, USA).

### 2.4 CD4 Proliferation Assay in Vivo

CFSE-labeled TCR-HA CD4^+^ T-cells were adoptively transferred to recipient BALB/c mice (2.5–3.5 × 10^6^ cells per mouse) by intravenous tail vein injection in a total volume of 200 μL. Recipient mice were immunized with VLPs (diluted in sterile PBS) by intravenous or subcutaneous injection in a final concentration of 200 ng of p24 per mouse. Inflexal^®^ V season 2011/2012 (Baxter International Inc., Deerfield, IL, USA) was applied subcutaneously in a volume of 100 μL per mouse. Three days after VLP injections, recipient mice were sacrificed, spleen and draining LN were removed and CFSE dilution in CD4^+^ CFSE^+^ cells was analyzed by flow cytometry.

### 2.5. CD4 Proliferation Assay in Vitro

Naive untouched CD4^+^ T-cells were isolated from TCR-HA and BALB/c mice, labeled with CFSE and co-cultured together with splenic DCs (10:1) for 64 hours at 37 °C in the presence of different VLPs at a concentration of 50 ng p24/mL. After incubation, the cells were stained with anti-CD3 V500 (BD Bioscience, Heidelberg, Germany) and anti-CD4 antigen-presenting cells (APC) (BD Bioscience) antibodies. CFSE dilution in CD3^+^ CD4^+^ was analyzed by flow cytometry.

### 2.6. B-Cell Proliferation and Expansion Assay in Vivo

SW-HEL B-cells (labeled or unlabeled with CFSE) were transferred to recipient BL6 mice (3–5 × 10^6^ cells per mouse) by intravenous injection. On the same day, recipient mice were immunized with HEL-VLPs by intravenous or by subcutaneous injection in a final concentration of 100 ng of HEL per mouse. Mice having received CFSE-labeled B-cells were sacrificed three days after the VLP injections; spleen and draining LNs were removed and stained with HEL-Alexa647 and with anti-B220 APC-eFlour (eBioscience, San Diego, CA, USA) antibody for HEL-transgenic B-cell receptor (BCR) [12] and total B-cells, respectively. CFSE dilution in HEL^+^ B220^+^ CFSE^+^ cells was analyzed with flow cytometry. Seven days after the transfer of unlabeled SW-HEL B-cells, spleens and LNs were also recovered, and SW-HEL B-cells cells were stained with HEL-Alexa647 and anti-B220 antibodies. Proliferation and expansion of HEL^+^ B220^+^ cells was analyzed by flow cytometry.

### 2.7. B-Cell Proliferation in Vitro

Freshly isolated splenic CD11c^+^ DCs (BL6) and CFSE-labeled B-cells (from SW-HEL and BL6 mice) were separately incubated for 2 h in the presence of either HEL-VLP or monomeric HEL in a final concentration of 100 ng/mL of HEL. After extensive washing, DC and B-cells were plated together (1:1) in U-bottom 96-well plates at a final density of 1 × 10^6^ cells/well and co-cultured for 3 days. Thereafter, cells were stained with anti-CD11c APC (BD Bioscience) and anti-B220 APC-eFlour (eBioscience) antibodies. Proliferation of CD11c^-^ 220^+^ B-cells was analyzed by flow cytometry.

## 3. Results

### 3.1. Adaptation of a TCR-Transgenic Mouse Model for the Activation of CD4^+^ T-Helper Cells by VLP-Derived Epitopes

**Figure 1:**
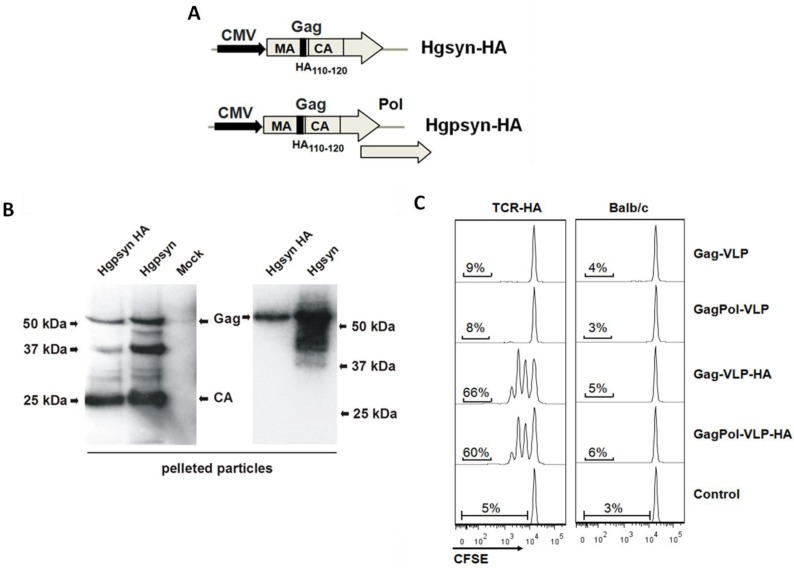
Design, production and characterization of HIV-virus-like particles (VLPs) containing an HA epitope. (**A**) Schematic representation of the Hgpsyn-HA and Hgsyn-HA expression cassettes. CMV, immediate early promoter of human cytomegalovirus; MA, matrix; CA, capsid; Pol, polymerase. HA_110-120_, influenza virus hemagglutinin epitope. (**B**) HEK293T cells were transfected with Hgpsyn, Hgpsyn-HA, Hgsyn or Hgsyn-HA. VLPs were recovered from the supernatants of transfected cells by ultracentrifugation through a sucrose cushion and analyzed by western blot with an antibody against capsid. (**C**) Naive untouched CD4^+^ T-cells were isolated from TCR-HA and BALB/c mice, labeled with CFSE and co-cultured with splenic DC at a 10:1 ratio for 64 h at 37 °C in the presence of the indicated VLPs (50 ng of capsid/mL). VLPs were prepared by co-transfection of an Env expression plasmid with one of the Gag or GapPol expression plasmids, as described in Table 1. After incubation, the cells were stained with anti-CD3 and anti-CD4 antibodies, and the CFSE fluorescence intensity of the CD3^+^ CD4^+^ cells is shown.

Due to their low frequency, it is difficult to explore the activation and differentiation of antigen-specific T- and B-cells during the first few days after antigen exposure. T- and B-cell receptor transgenic mouse models are better suited to explore these early events. Since TCR-transgenic mouse models for HIV proteins are not available, we adapted the TCR-HA mouse model by incorporating a heterologous epitope into HIV-VLPs. The α/β-TCR-transgenic CD4^+^ T-cells from TCR-HA mice recognize the hemagglutinin HA_110-120_ epitope of influenza virus on the H-2Ed allele of MHC-II [14]. To generate VLPs containing this HA epitope, the coding sequence for the HA_110-120_ epitope was inserted in frame between the matrix and capsid open reading frames of codon-optimized HIV-Gag and HIV-GagPol expression plasmids (Figure 1A). To confirm particle formation, both plasmids were transiently transfected into 293T cells, and VLPs were partially purified by pelleting through a 20% sucrose cushion. Western blot analysis with a capsid-specific antibody revealed the expected Gag protein (Figure 1B). No differences in the size of the Gag protein of HA containing VLPs and wild-type VLPs were observed, probably due to the small size of the 10-amino acid HA epitope. In the case of VLPs encoded by the HIV-GagPol expression plasmids, capsid-containing cleavage products could also be identified, indicating proper processing by the viral protease.

Since little is known about how Gag maturation in the HIV particles affects its processing and MHC class II-mediated presentation by professional antigen-presenting cells (APC), the presentation efficacy of the HA epitope from mature GagPol and immature Gag particles were determined by an *in vitro* T-cell proliferation assay. The HIV-VLPs were prepared as described above by co-transfection of the HIV-GagPol and HIV-Gag expression plasmids with or without the coding sequence for the HA epitope together with an HIV-Env expression plasmid. Naive CFSE-labeled CD4^+^ T-cells from TCR-HA or wild-type (wt) BALB/c mice were co-cultured with freshly isolated splenic dendritic cells (DCs) in the presence of HIV-VLPs with or without the HA_110–120_ epitope. While VLPs lacking the HA do not induce a proliferative response, Gag and GagPol VLPs containing the HA epitope selectively trigger the proliferation of TCR-HA, but not wild-type CD4^+^ T-cells (Figure 1C). Approximately 85% of the TCR-HA CD4^+^ T-cells do not express the transgenic TCR and, therefore, do not proliferate, even if exposed to VLPs containing the HA epitope. This internal control further excludes unspecific stimulatory effects of our VLP preparations. Thus, the VLPs containing the HA epitope seem to be well suited for the analysis of the early proliferative responses of TCR-HA CD4^+^ T-cells to VLP immunizations.

**Table 1:** Lentiviral VLP preparations used in the study.

Abbreviations	Envelope Proteins	Core Proteins
Gag-VLP	HIV-Env	HIV-Gag
GagPol-VLP	HIV-Env	HIV-Gag/Pol
Gag-VLP-HA	HIV-Env	HIV-Gag with HA_110-120_
GagPol-VLP-HA	HIV-Env	HIV-Gag/Pol with HA_110-120_
HEL-VLP	Membrane-anchored HEL	HIV-Gag/Pol

### 3.2. Proliferative Response of Naive CD4^+^ T-Cells to VLP-Derived Epitopes in Vivo

To analyze the proliferative response of cognate naive CD4^+^ T-cells to epitopes derived from HIV VLPs *in vivo*, we adoptively transferred CFSE-labeled CD4^+^ T-cells isolated from TCR-HA mice into naive BALB/c mice. The recipient animals were then immunized with HIV-GagPol VLPs containing the HA epitope by the intravenous and subcutaneous route. As a positive control, mice were also immunized with Inflexal^®^ V, an adjuvanted virosomal influenza vaccine containing the hemagglutinin of the pandemic H1N1 influenza virus from December, 2011.

**Figure 2:**
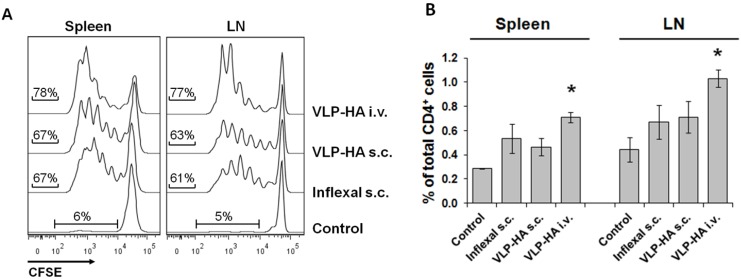
Proliferative response of naive cognate CD4^+^ T-cells after s.c. and i.v. immunization. Naive untouched CD4^+^ T-cells were isolated from TCR-HA mice labeled with CFSE and adoptively transferred into recipient BALB/c mice (2.5 × 10^6^ cells per mouse). Four hours later, the recipient mice were immunized with GagPol-VLP-HA (s.c. or i.v.) or with Inflexal^®^ V s.c. (**A**) Proliferation of the transferred CD4^+^ T-cells from the spleen and draining lymph nodes (LNs) was analyzed 72 h after immunization. The CFSE fluorescence intensity of CD3^+^ CD4^+^ is shown. (**B**) Expansion of transferred T-cells is evident by the increase in the percentage of CD4^+^ CFSE^+^ T-cells from total CD4^+^ lymphocytes in the spleen and lymph nodes. The histogram gives the mean of one experiment ± the standard deviation (*n* = 3). * Significant difference for the group in comparison to the other groups (*p* < 0.05; Student’s *t*-test). The experiment was repeated two more times with Gag-VLP-HA with similar results.

The proliferative response was determined in the spleens and the inguinal lymph nodes draining the subcutaneous immunization site. The intravenous route induced the strongest proliferative response and expansion of the CFSE-labelled TCR-HA T-cells (Figure 2A,B). The proliferative response in spleens and draining LNs was comparable within each immunization group (Figure 2A). In addition, Gag VLPs induced a very similar TCR-HA CD4^+^ T-cell proliferative response (data not shown). This indicates that maturation of the VLP by proteolytic processing of Gag had little or no influence on processing and presentation of the HA epitope.

### 3.3. Proliferation and Expansion of Naive Cognate B-Cells after VLP Immunization

**Figure 3:**
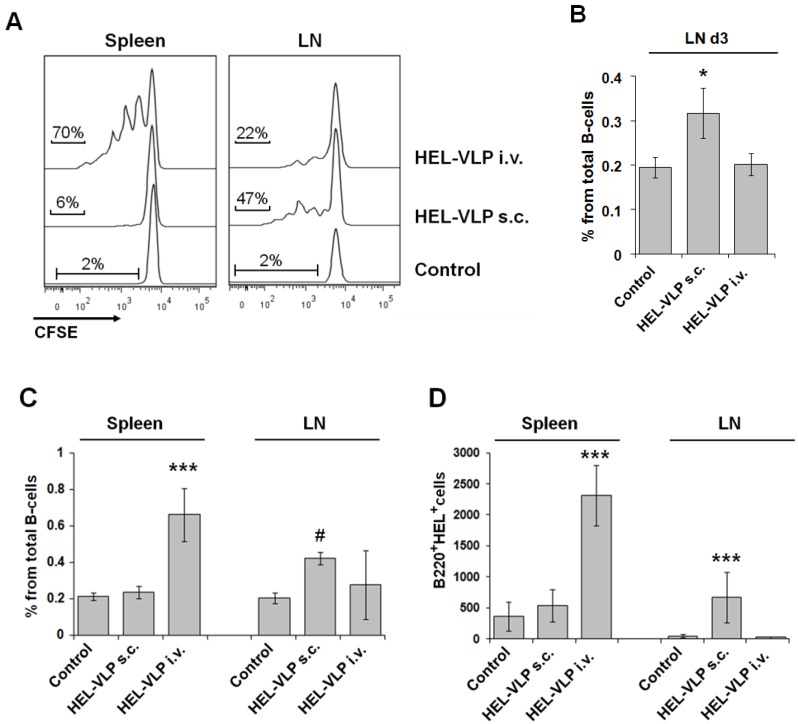
Proliferative response and expansion of naive cognate B-cells after s.c. and i.v. immunization. Naive untouched B-cells were isolated from SW-HEL mice, labeled with CFSE and adoptively transferred into recipient BL6 mice (5 × 10^6^ cells per mouse). Four hours later, the recipient mice were immunized with HEL-VLP (s.c. or i.v.). (**A**) Three days later, spleen and LN cells were stained with Alexa647-conjugated HEL and anti-B220 antibodies. The CFSE fluorescence intensity of HEL^+^ B220^+^ cells is shown. (**B**) The expansion of HEL^+^ B220^+^ CFSE^+^ B-cells in draining LN on Day 3 is evident by the increase in their percentage of the total B220^+^ cells. The histogram gives the mean ± the standard deviation of one representative experiment (*n* = 4). Two independent experiments with three to four mice were performed. * Significant difference for the group comparison to the other groups (*p* < 0.05; Student’s *t*-test). (**C**, **D**) For the analyses of B-cell expansion on Day 7, non-labeled SW-HEL B-cells were transferred. Seven days after VLP injection, cells were stained with Alexa647-conjugated HEL and anti-B220 antibodies, and the expansion of HEL^+^ B220^+^ cells is presented as the percentage of the total B220^+^ cells (**C**) and as their absolute numbers in the cell preparations from spleen and lymph nodes (**D**). The histograms represent the mean within one experiment ± the standard deviation (*n* = 4). *** Significantly different from the other groups (*p* < 0.01; Student’s *t*-test). ^#^ Significantly different from the control group (*p* < 0.01; Student’s *t*-test).

Previously, we had shown that HIV- and SIV-VLPs can induce a primary humoral immune response to Env in wild-type mice after subcutaneous immunization, even in the absence of adjuvants [3,10,15]. In addition, SIV-VLPs induced secondary antibody responses after i.v. delivery in a T-cell-independent manner [3]. However, the frequency of naive B-cells recognizing a particular antigen normally is rather low, making the phenotypic analysis of antigen-specific B-cells in wild-type animals rather complicated. To determine the antigen-specific proliferative response of B-cells to the surface protein of VLPs, we employed the B-cell receptor (BCR)-transgenic SW-HEL mouse model [12]. The B-cells of these mice express a transgenic BCR recognizing hen egg lysozyme and can mature and proliferate without any obvious abnormality [16]. To make VLPs with a cognate envelope-embedded protein, the HIV-Env protein of GagPol VLPs was replaced by membrane-anchored HEL [12], resulting in HEL-VLP (Table 1). The B-cells from naive SW-HEL mice were labeled with CFSE and adoptively transferred into naive recipient BL6 mice, which were then immunized with equal amounts of HEL-VLP by the i.v. or s.c. route. In contrast to the proliferative response of CD4^+^ T-cells, the proliferative response of the HEL^+^ B-cells was strongly dependent on the route of administration (Figure 3A). Intravenous application of HEL-VLP induced massive proliferation of HEL^+^ B-cells in the spleen, but not in the LN. At the same time, subcutaneous VLP immunization induced moderate proliferative response in draining LNs only (Figure 3A,B).

Although proliferation of naive Ag-specific B-cells was not detected in the spleen three days after s.c. VLP immunization, splenic B-cells may also be stimulated by antigen captured and presented by migratory or resident APC (DCs, macrophages, follicular DCs) (reviewed in [8]). Once T-cell help has developed, this type of activation may initiate germinal center reaction starting with the massive expansion of antigen-specific B-cells (reviewed in [8]). Therefore, we also analyzed the expansion of HEL^+^ B-cells in spleens and LN on Day 7 after primary VLP immunizations (Figure 3C,D). A significant increase in both the percentages and absolute numbers of HEL^+^ B-cells in the spleen was detected only after intravenous immunization with HEL-VLP. At the same time, the expansion of HEL^+^ B-cells in the draining LNs together with a general LN enlargement was only observed after s.c. immunization (Figure 3C,D).

### 3.4. Efficacy of B-Cell Activation by DC Mediated Transfer of VLP-Associated Surface Antigen in Vitro

**Figure 4:**
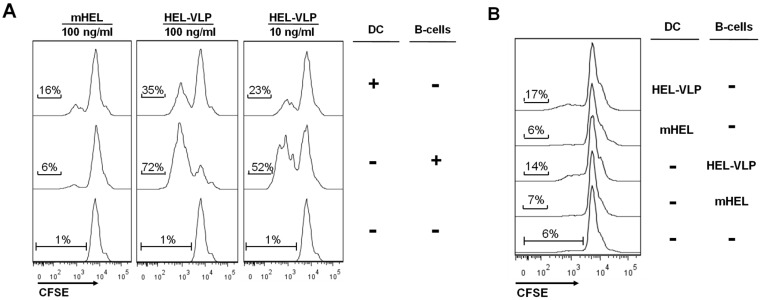
Transfer of particulate and soluble HEL by DC to cognate B-cells *in vitro*. Naive untouched B2-cells were isolated from (**A**) SW-HEL and (**B**) BL6 mice and labeled with CFSE. Freshly isolated splenic DC or B-cells were separately incubated for 2 h in the presence of either HEL-VLP or mHEL. After extensive washing, DC and B-cells either exposed to VLPs/mHEL as marked by the “+” sign or non-exposed as marked by the “-” sign were mixed together and co-cultured for three days. The experiment was performed three times independently of each other, and one representative experiment is shown.

DCs capture soluble antigens (Ag) *in vitro* and *in vivo* and may present this Ag in an unprocessed manner to naive B-cells, thereby initiating a specific Ab response [17]. DCs may also provide additional proliferation and survival signals to the B-cells [18,19], further supporting B-cell responses. Proliferation of cognate CD4^+^ T-cells in the spleen after s.c. VLP injection indicates that migratory DCs exposed to VLPs at the injection site reach the spleen and stimulate CD4^+^ T-cell responses (Figure 2), but not B-cell proliferation (Figure 3). We therefore explored the efficacy with which DCs present monomeric HEL (mHEL) and HEL incorporated into VLPs to SW-HEL B-cells. DCs or SW-HEL B-cells were first exposed to either mHEL or HEL-VLPs. After extensive washing, DCs and B-cells were co-cultured, and the proliferation of the SW-HEL B-cells was analyzed (Figure 4).

As observed previously [12], monomeric HEL is a rather poor inducer of HEL^+^ B-cell proliferation *in vitro*, while HEL-VLPs stimulate their efficient proliferation (Figure 4A). This proliferation is antigen-specific, since B-cells from non-BCR transgenic mice do not respond (Figure 4B). Exposure of DCs to monomeric HEL enhanced the proliferative response of SW-HEL B-cells in comparison to direct stimulation of the B-cell with the protein (Figure 4A). However, when the same amount of HEL or a 10-fold lower amount were presented on the surface of VLPs, the situation was reversed. The proliferative response of cognate B-cells after direct stimulation with lentiviral VLPs was stronger than the response mediated by DCs exposed to the VLPs (Figure 4A).

## 4. Discussion

Although both T- and B-cells are involved in the adoptive response to the same pathogens, their antigen recognition is based on two entirely different mechanisms.

The cognate interaction of CD4^+^ T-cells with activated DCs presenting processed antigen on MHC-II complexes and reciprocal co-stimulatory molecules initiates CD4^+^ activation. This initial activation results in extensive clonal expansion (proliferation) of antigen-specific CD4^+^ T-cells (reviewed in [9]). Previously, it was shown that HIV-VLPs produced in yeast were internalized by human DCs, leading to the activation of Gag-specific T-cells in HIV-infected individuals, but not in healthy donors [20]. In addition, DCs loaded with HIV-VLPs produced in a Baculovirus expression system were able to induce a primary and secondary response in autologous human CD4^+^ T-cells *ex vivo* [21]. However, the reported stimulatory effects of these VLPs may have been influenced by the presence of compounds derived from the producer cells. Trace amounts of the yeast cell wall component, zymosan, remaining in the VLP preparation even after treatment by zymolase, may have been recognized by TLR2 [20], reviewed in [22]. Baculo-derived VLPs harbor, in addition to substantial amounts of insect cell-derived lipids, nucleic acids and proteins, high levels of baculovirus envelope proteins, which may direct the fusion of the VLPs with the cell membrane of the antigen-presenting cells (reviewed in [22]). In contrast, our HIV-VLP production is based on the eukaryotic HEK 293T cell line. In the VLP preparations, endotoxin levels were extremely low, and general contamination with innate stimuli was below the threshold level for polyclonal activation of T-cells (Figure 1C) and B-cells (Figure 4B). At the same time, subcutaneous injection of VLPs containing the HA epitope without any adjuvant induced a comparable primary antigen-specific CD4^+^ T-cell proliferative response as the adjuvanted Influenza vaccine, Inflexal^®^ V (Figure 2). We hypothesize that VLP-derived peptides are presented at high densities by those APCs that have taken-up an entire VLPs, since each VLP contains approximately 2000 molecules of Gag. A high density of peptide-loaded MHC molecules may overcome the requirement of an adjuvant. Our previous experiments in wild-type mice also revealed that similar HIV-and SIV-VLPs stimulated reasonable levels of Env antibody responses in the absence of adjuvants [3,10], indicating that at least the B-cell response to VLPs is not limited to the initial proliferative effect observed in the present study.

In contrast to CD4^+^ T-cells, the initiation of adoptive humoral immune response requires the encountering of the cognate B-cells with non-degraded antigen in B-areas of secondary lymphoid organs. After s.c. injection, the VLPs may enter lymphoid organs either as free lymph-borne particles or may be actively transported to the lymphoid organs by migrating DCs from the injection site (reviewed in [6,7,8]). After i.v. injection, the VLPs either reach the lymphoid organs via circulation or are also transported in by migratory DCs. Our *in vitro* observation, that monomeric HEL stimulates B-cell proliferation more efficiently if the antigen is first captured by DCs, while for the VLPs, direct exposure of B-cells is more efficient, suggests that the optimal delivery route for VLPs may also differ from non-particulate antigens. The direct comparison of the subcutaneous and intravenous delivery routes revealed important differences in the B- and T-cell proliferative response to VLPs. While proliferation of cognate CD4^+^ T-cells in lymph nodes and spleens was observed for both routes, proliferation of HEL-specific splenic B-cells was only observed after intravenous VLP immunization. On the contrary, the proliferation of HEL-specific B-cells in lymph nodes was more efficient after subcutaneous than intravenous immunization. Although HIV/SIV are blood-borne pathogens, i.v. injections have rarely been considered as a potential immunization route for VLPs. From the present study, it is not possible to predict which of the VLP delivery routes is best suited to induce protective antibody responses against HIV. Although the proliferative response is dependent on proper antigen presentation and can be considered to be an early marker of immunogenicity, the protective efficacy of the antibody response is affected by more complex immunoregulatory networks, modulating, for example, IgG subtype usage, B-cell memory, the duration of antibody responses and antibody affinity. However, the differences in the B-cell proliferative responses after intravenous and subcutaneous immunization with VLPs argue for further studies on the quality of immune responses after intravenous administration of VLPs, an approach rarely pursued in the past.
